# Current Status and Trends of Low-Temperature Steel Used in Polar Regions

**DOI:** 10.3390/ma17133117

**Published:** 2024-06-25

**Authors:** Qiaoling Xiao, Yaozhou Xie, Feng Hu, Chengyang Hu

**Affiliations:** Collaborative Innovation Center for Advanced Steels, Wuhan University of Science and Technology, Wuhan 430081, China; 1401051@wust.edu.cn (Q.X.); hufeng@wust.edu.cn (F.H.)

**Keywords:** polar steel, low-temperature steel, low temperature toughness, corrosion, anticorrosion

## Abstract

The desire to explore the natural resources and geopolitical patterns of the North and South Poles has significantly increased the interest of experts and researchers in the development and utilization of the polar regions. In this article, we comprehensively analyzed the current state of the development of polar low-temperature steel around the world. We highlighted the challenges that must be addressed in the ongoing development efforts and summarized the expected future trends in this field. The main theme of this article involves the challenges encountered in polar environments primarily caused by the low-temperature toughness and seawater corrosion of marine steel.

## 1. Introduction

The combined surface area of the north and south polar regions exceeds 35 million km^2^, which is approximately 2.5 times the total land area of China. The scientific exploration of the north and south poles has a rich history, spanning over a century. China is an active participant in polar research (Mainly in Antarctica) and commenced its official scientific investigations in the 1880s [1]. Reports on the key findings from the exploration programs highlighted the abundance of energy, mineral, and biological resources in the polar regions, specifically in the north and south poles. For example, the Arctic region has approximately 13% of the global undiscovered oil reserves, 30% of the untapped natural gas reserves, and 9% of the coal resources available worldwide [2]. The polar regions have various minerals, such as gold, copper, iron, lead, platinum, nickel, and zinc. [3] However, the environmental conditions in the polar regions are harsh, and there are various challenges, such as low temperatures, sea ice, icebergs, snowstorms, fragile ecological environment, polar night, and poor visibility. Hence, selecting the correct type of steel to be used in the polar region is important for making maritime transportation safer, reducing the duration of the voyage and decreasing the associated transportation costs.

For example, ships are the main mode of transport in the polar region. Thus, materials that can withstand low temperatures need to be developed for constructing these special ships that operate in the polar region. These materials include low-temperature high-strength steel for ships, high-strength steel for cryogenic vessels, non-metallic sealing materials suitable for low temperatures, coating materials that prevent ice formation, lightweight thermal insulation materials, and low-temperature cable materials [4]. Polar steel must retain its strength, toughness [5,6,7,8], and weldability [9,10,11,12,13] under extremely low temperatures. It must also be resistant to corrosion-induced degradation and loss of structural integrity. Simultaneously, icing concerns need to be addressed to make the structure safe and dependable. When making decisions regarding the selection of steel grades, the delicate ecological environment of the polar region needs to be accounted for.

## 2. Development and Research Status of Polar Low-Temperature Steel

### 2.1. Polar Low-Temperature Steel Involved in Key Engineering Projects

Russia has made the greatest advancements in the development of polar steel, while other nations, including Japan, the United States, South Korea, and Finland, have also made progress in the development of low-temperature steel for polar ships [14]. Russia has manufactured a composite steel material that exhibits exceptional resistance to corrosion and has advantageous properties at low temperatures. The external surface of this material consists of stainless steel, while the internal layer consists of high-strength, low-temperature steel. The outer layer acts as a protective barrier against corrosion, while the inner layer provides structural reinforcement and mitigates the risk of brittle fracture under low-temperature conditions. The JFE Steel Corporation, located in Japan, implemented the HOP (Online Heat Treatment Process) process and the Super-OLAC (online ultra-fast accelerated cooling) device to produce steel plates [15]. These processes facilitated rapid uniform cooling of the steel plate, approaching the theoretical limit of water cooling. When the carbon equivalent is low, a uniform and high-performance microstructure can be obtained. This can enhance the weldability of low-temperature steel and increase the reliability of ship structures. JFE has also developed a type of stainless steel, known as JSL310Mo, which exhibits high resistance to corrosion caused by seawater. This alloy is primarily composed of 25% chromium, 4.5% molybdenum, and 0.2% nitrogen. The alloy also contains nickel and trace amounts of boron when JSL310Mo is used as the coating material.

In 2016, Baosteel Co. Ltd. (Shanghai, China) spearheaded China’s key national research and development project titled “Marine Steel in Extremely Cold and Ultra-low Temperature Environment and its Application.” This project focused on conducting fundamental research on strengthening and toughening mechanisms, as well as on fracture mechanisms of cryogenic steel [16]. It investigated the design of composition processes for ultra-low temperature marine steel plates, investigated key technologies related to manufacturing processes, and conducted research on the application of welding materials. The joint laboratory titled “Manufacture and Corrosion Control of Steel Materials in Marine Extreme Environment,” established in 2017 by the College of Marine Materials Science and Engineering of Shanghai Maritime University and Baosteel Co., Ltd., Shanghai, China, has made progress in its research [17]. In 2017, 20 tons of cryogenic steel was used for the internal modification of the scientific research vessel “Xuelong”. In 2019, approximately 1000 tons of polar special cryogenic steel was used for developing another vessel known as “Xuelong 2”. The steel materials for polar low-temperature environments were developed through a collaboration between Wuhan University of Science and Technology and Nanjing Iron and Steel Co. Ltd., (NISCO), Nanjing, China. The program aimed to meet the specific requirements of polar equipment. An industrial trial was conducted for producing EH36 (the shipbuilding steel plate refers to the carbon and alloy steel plate used in offshore and marine constructions; common grades are A, B, D, E, AH32/36/40, DH32/36/40, and EH32/36/40, which have different levels of strength), which has stringent specifications, and a production output exceeding 1000 tons was achieved.

### 2.2. Studies on the Strength and Toughness of Polar Low-Temperature Steel

Low-temperature brittle failure in polar equipment under static load conditions is often associated with low-temperature toughness. The concept of “low-temperature toughness” refers to a material’s ability to withstand plastic deformation and fracture under impact loads at low temperatures. The low-temperature toughness of materials is usually characterized by the steel strength index (yield strength $f_{y}$ and tensile strength $f_{u}$), the Charpy impact energy $A_{kv}$, and the material’s fracture toughness index (crack tip open displacement, CTOD). Parameters such as the stress concentration, residual stress, and cross-sectional size of marine equipment affect the low-temperature toughness of materials. However, the requirement of a low-temperature mechanical structure only for cryogenic equipment is one-sided [18]. To mitigate the risk of equipment failure due to low-temperature brittleness, multiple aspects, such as material properties, structural design, and manufacturing processes, need to be addressed [19,20,21,22,23,24]. A comprehensive examination of the effect of various factors on material toughness at low temperatures is necessary.

A characteristic drop in temperature can lead to a rapid shift from a ductile to a brittle state within a specific temperature range, known as the ductile-to-brittle transition temperature (DBTT) [25,26,27,28,29]. Research indicates that impurities like N, O, and S can lead to a significant increase in the ductile-to-brittle transition temperature [28]. The inclusion of certain alloying elements can lower the ductile-to-brittle transition temperature [29,30]. Research suggests that the addition of alloying elements can trap O atoms within the crystal lattice, thus reducing their concentration at grain boundaries, enhancing grain boundary strength, and contributing to material toughening [31]. The factors that affect the ductile brittle transition temperature are not only the purity and impurity elements described above but also the grain size [32,33], dislocations [34,35,36], and second phase [37,38]. Although numerous researchers have offered explanations for each of these factors, a comprehensive mechanism that encompasses all of these variables is still lacking. Therefore, further research is essential to establish a linkage between macroscopic and microscopic levels, develop effective theories, and construct a systematic and comprehensive physical model for the DBTT.

Pipeline steel is extensively utilized for the long-distance transportation of oil and natural gas in polar regions [39,40,41]. As development in polar regions progresses, pipelines are subjected to increasingly severe environmental conditions. Typical grades of pipeline steel include X70, X90, and X100 (in the API (American Petroleum Institute) Spec 5L standard [42], X denotes pipeline steel, with a strength grade of 70 and kpsi as the unit). As the grade of pipeline steel increases, so does its strength. However, enhancing toughness becomes progressively challenging with increasing strength. Consequently, it is crucial to improve the toughness of pipeline steel while maintaining its strength. The microstructure plays a significant role in determining the mechanical properties of pipeline steel.

**Figure 1:**
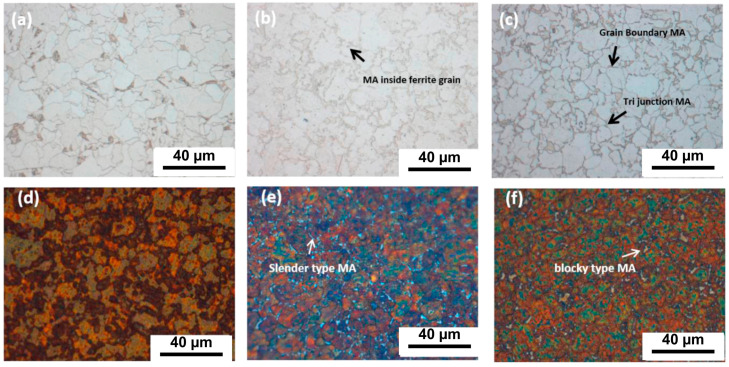
Microstructure after heat treatment (710 °C); (**a**,**d**) furnaced-cooled sample; (**b**,**e**) air-cooled sample; and (**c**,**f**) water-cooled sample revealed using nital and Lepera etchants, respectively [43].

**Figure 2:**
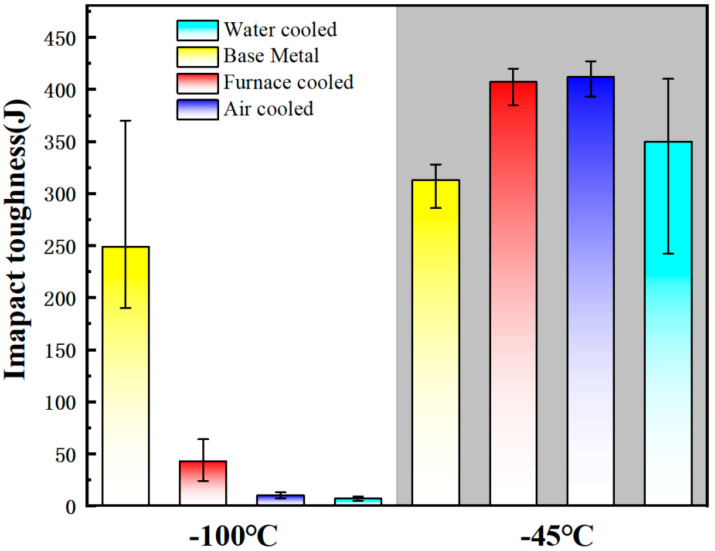
Impact toughness energy comparison at −100 °C and −45 °C [43].

Nuda et al. [43] conducted a study to investigate the influence of different cooling rates on the content of martensite austenite (MA) microconstituent in X80 pipeline steel. As depicted in Figure 1, the heat-treated sample that was cooled in the furnace displays a structure consisting of ferrite and degenerated pearlite (DP) (Figure 1a). However, samples cooled using air and water exhibit small MA islands at the triple junctions, grain boundaries, and even within the ferrite grains (Figure 1b,c). The use of Lepera etchant on the furnace-cooled sample does not reveal any white structure, indicating the absence of MA. In contrast, the samples cooled using air and water display numerous white islands in their structure (Figure 1e,f). Both samples exhibit a slender (aspect ratio of 4:1) and block-like structure. Nonetheless, the microstructure of the water-cooled sample primarily consists of blocky MA. This suggests that increasing the cooling rate promotes the formation of MA, while its morphology changes from elongated to blocky. Charpy impact toughness tests were performed on the original base material and the heat-treated samples (furnaced-cooled, air-cooled, and water-cooled) at temperatures of −100 °C and −45 °C to evaluate their fracture behavior. Figure 2 illustrates that at −45 °C, the furnace-cooled sample and the air-cooled sample exhibit similar average impact toughness performance, whereas the water-cooled and the original samples demonstrate significantly lower average impact toughness performance. At −100 °C, the average impact toughness of the original base material is 250 J; the furnace-cooled sample measures 42 J; and the air-cooled and water-cooled samples record 12 J and 6 J, respectively. In general, finer grains can enhance the low-temperature toughness of materials. However, the presence of MA islands can have detrimental effects on toughness [44,45]. This is because MA islands are considered brittle structures, and the impact toughness of MA islands formed along grain boundaries is reduced.

Microalloying is widely employed as a common method to enhance the low-temperature toughness of polar steel [46]. The addition of Ni improves the low-temperature toughness of steel and provides high corrosion resistance to acid and alkali. It is particularly effective in stainless steel containing Cr, where it stabilizes the passivation film in austenitic steel, resulting in excellent corrosion resistance in acidic and alkaline environments. However, practical considerations need to be taken into account regarding the scarcity of Ni resources and the reasonable control of their addition amount. The low cost of manganese and its suitability for industrial mass production using conventional processes make the Fe-Mn-C alloy with a fine grain structure an economically advantageous steel product compared to high-entropy alloys with high-temperature and low-temperature toughness. In a study conducted by Wang et al. [47], high-manganese and low-carbon steel with exceptionally high and low-temperature toughness was fabricated. By combining these austenite-stabilizing elements with an appropriate fine grain size, the inverse temperature dependence of toughness was achieved, and a low-temperature toughness exceeding 450 J (a Charpy impact energy of 453 J is achieved at liquid nitrogen temperatures, which is about four to five times that of conventional cryogenic austenitic steels) was recorded in the fine-grained Fe-30Mn-0.11C steel.

**Figure 3:**
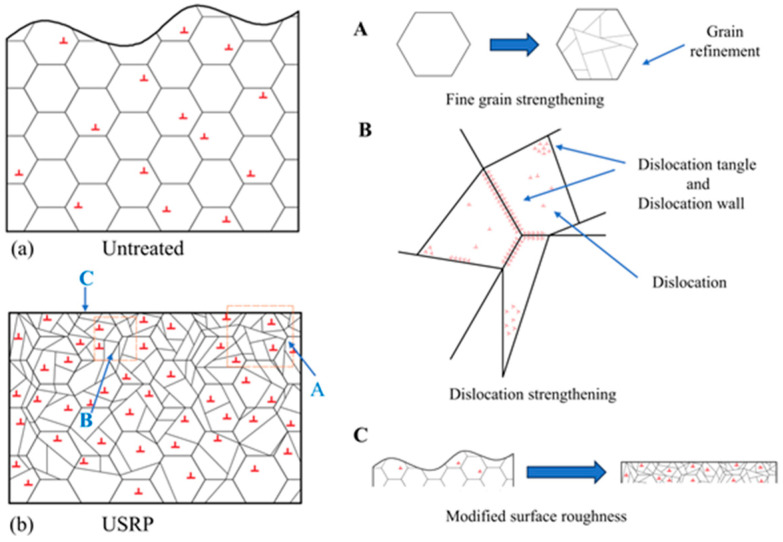
A schematic diagram of the mechanism by which USRP strengthens the low-temperature mechanical properties of FH36 marine steel [48]. The surface grains of FH36 marine steel exhibited a uniform distribution before using the USRP approach and a few dislocations were also randomly dispersed within the grains (**a**). After utilizing USRP, the surface grains of FH36 marine steel underwent fracture due to exposure to high-frequency vibrations and dynamic impact (**b**).

The appropriate process can improve the low-temperature toughness of polar materials. The traditional rolling and annealing process can improve the low-temperature toughness of steel. Xu et al. [48] performed the ultrasonic surface rolling process (USRP) to enhance the low-temperature mechanical properties of FH36 (a high strength shipbuilding steel plate is composed of four quality levels (A, D, E, and F) and three strength levels (32, 36, and 40) combined) marine steel. The technique was implemented to improve the overall service performance of marine steel in low-temperature environments, specifically in regions with extremely low temperatures, such as polar regions. The temperature range considered in that study ranged from −30 °C to −60 °C. The findings indicated that the surface integrity of FH36 marine steel underwent reorganization as a result of plastic deformation induced by USRP. The average grain diameter of the surface decreased from 10.92 µm to 5.86 µm, accompanied by a substantial increase in the quantity of nano-sized grains. Consequently, a nanostructured surface layer was formed. The surface roughness decreased significantly (Figure 3b(C)), which resulted in a Ra value below 0.074 µm. A schematic diagram illustrating the influence of the USRP technique on the low-temperature mechanical properties of FH36 marine steel is shown in Figure 3. The surface grains of FH36 marine steel exhibited a uniform distribution before using the USRP approach (Figure 3a). A few dislocations were also randomly dispersed within the grains. After utilizing USRP, the surface grains of FH36 marine steel underwent fracture due to exposure to high-frequency vibrations and dynamic impact (Figure 3b). The process of grain refinement led to the formation of a surface layer composed of nanostructures, which in turn promoted the strengthening of fine grains (illustrated in Figure 3b(A)). The simultaneous application of severe plastic deformation increased the density of dislocations and resulted in dislocation strengthening. This strengthening effect arose from the entanglement of dislocations caused by the presence of dislocation walls within grains and at grain boundaries (Figure 3b(B)).

**Figure 4:**
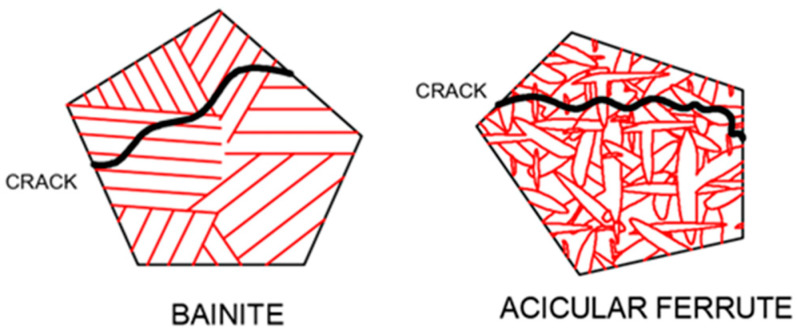
Propagation diagram of cleavage crack in bainite and acicular ferrite.

**Figure 5:**
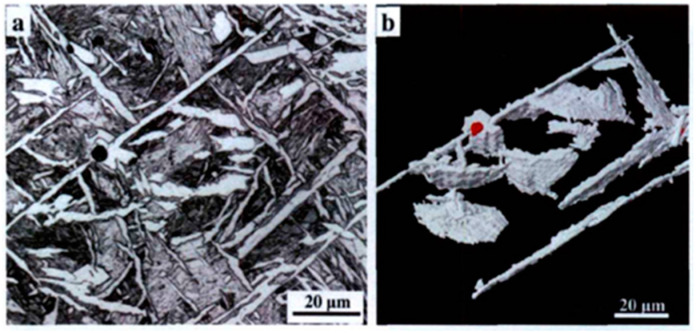
The morphology of acicular ferrite nucleated on inclusions in low-carbon steel: (**a**) two-dimensional morphology and (**b**) three-dimensional morphology (white). The color represents acicular ferrite [50].

As external stress continues to increase, microcracks develop within the material and gradually propagate. The presence of large-angle grain boundaries indicates a significant disparity in the positions of adjacent grains. This can alter the direction of crack propagation and impede its progress, thereby making it challenging for cracks to propagate and enhancing toughness. Conversely, when cracks encounter small-angle grain boundaries, they can easily pass through and continue propagating in their original direction, which hinders effective obstruction [49]. When the crack encounters a low-angle grain boundary, it can directly pass through without changing the original direction of expansion; under such conditions, it cannot be effectively hindered. The acicular ferrite structure is composed of interlaced laths, and the high-angle grain boundary density has high toughness. A schematic diagram of cleavage crack propagation in bainite and acicular ferrite is shown in Figure 4. Bainite is composed of parallel laths. The density of high-angle grain boundaries is small, and cracks can easily propagate throughout the entire lath bundle, which cannot be effectively hindered and has poor toughness. The interlaced ferrite laths in acicular ferrite make the crack tortuously expand, effectively hinder crack propagation, and enhance toughness. The two-dimensional morphology and the corresponding three-dimensional morphology of acicular ferrite after heat treatment of low-alloy steel, observed by our research group [50], are shown in Figure 5. Acicular ferrite laths were found to nucleate from the inclusions and grow up to the surrounding radiation; the three-dimensional morphology was found to be plate-like. It is difficult to obtain the profile of the two-dimensional observation of a sample in a single acicular ferrite width because under the microscope it has a needle-like presentation.

Ferrite and acicular ferrite multiphase steels not only have a low yield ratio and high uniform elongation but also have high low-temperature toughness. Although some researchers studied and developed ferrite and acicular ferrite multiphase steels, they did not consider the extremely low-temperature environment [51,52]. For example, the room temperature in the Arctic region can be as low as −70 °C, requiring steel to have excellent low-temperature toughness. For ferrite steel, the refinement of ferrite in a multiphase structure can effectively reduce the ductile-to-brittle transition temperature [53]. For hot-rolled plates, fine ferrite can be formed by the deformation between the temperature between Ar3 and Ae3 [54]. Therefore, the multiphase structure of ferrite and acicular ferrite can be formed by the TMCP (Thermo-Mechanical Control Process) process to enhance the low-temperature toughness of steel plates.

The purity of steel is a critical factor influencing low-temperature toughness [55,56]. The ductile-to-brittle transition temperature of steel rises with an increase in the content of interstitial solute elements. These interstitial solute elements not only cause lattice distortion in the matrix but also tend to accumulate near dislocation sites and interact with dislocations, resulting in the formation of Coriolis gas clusters. This, in turn, has a detrimental effect on the low-temperature toughness of steel. Sulfides or oxides in steel are prone to becoming sources of cracks during low-temperature impact. Therefore, ensuring the purity of steel is beneficial for improving the low-temperature toughness of steel [57,58,59,60,61]. And the composition and quantity of inclusions, as well as the presence of impurity elements, can have a serious impact on the low-temperature toughness of steel. Therefore, in order to improve the toughness, it is necessary to adjust the morphology of inclusions in the smelting process and minimize the introduction of harmful elements in the production of pipeline steel.

Therefore, to enhance toughness, the morphology of inclusions needs to be regulated during the smelting procedure, and the introduction of detrimental elements needs to be minimized while producing pipeline steel. The results of theoretical calculations and empirical investigations showed that introducing magnesium or calcium during the smelting process can alter the nature of inclusions in pipeline steel [62]. This treatment facilitates the conversion of alumina inclusions, which have detrimental effects on toughness, into spherical composite inclusions. Treatment with calcium can induce a transformation of a portion of MnS inclusions into CaS, which can mitigate the deleterious effects of MnS inclusions on the corrosion resistance of polar steel. Wang et al. [63] studied the characteristics, sources, and control methods of longitudinal beam-shaped B-type non-metallic inclusions in pipeline steel. They found that the strip-shaped B-type non-metallic inclusions in API X80 pipeline steel plate are mainly a CaO-Al_2_O_3_ system with a lower melting temperature. During the rolling process, these inclusions may also be elongated into longitudinal beam-type inclusions due to their soft CaO-Al_2_O_3_ centers. A new strategy is now used to control B-type inclusions in pipeline steel plates. The focus of control has shifted from removing low-melting point inclusions in the CaO-Al_2_O_3_ system after Ca treatment to removing inclusions, especially large inclusions, as much as possible before Ca treatment. After adopting this new strategy, the number of inclusions in RH refining (a steel-refining method designed and developed jointly by Ruhrstahl and Hereaeus) decreased, and the efficacy of Ca treatment increased. The inclusions found in the steel plate are all from the high-melting-point CaO-CaS series, and the severity of B-type inclusions decreased from “≤2.0” to “0” (ASTM (American Society of Testing Materials) E45 standard [64]).

The anisotropy of steel plates is a critical aspect of polar steel that cannot be ignored. Owing to the anisotropy induced during the rolling process, the fracture behavior of steel plates displays pronounced directional differences. This disparity may result in steel plates being more susceptible to fracture in certain directions and less so in others [65]. However, regarding the anisotropy of mechanical properties, numerous studies have drawn differing conclusions, ranging from the assertion that crystal texture has no impact on toughness anisotropy to the identification of specific, larger orientations [66,67,68,69,70,71]. Moreover, for operational workpieces, accurately measuring the mechanical properties in their necessary orientations via experiments proves challenging.

### 2.3. Corrosion Resistance of Polar Steel

Corrosion in polar environments experienced by steel materials is complex and unique. The three commonly used methods to enhance the corrosion resistance of polar steel materials are alloying, coating technology, and cathodic protection technology [72,73,74,75,76,77,78]. These approaches are used to reduce the corrosion of polar steel materials. Alloying is an important technique used to enhance the mechanical properties, specifically the strength and toughness, of materials.

#### 2.3.1. Microalloying

The alloying of materials commonly involves the addition of elements such as Ni, Mn, Nb, Cr, Mo, and N to steel. The application of rare earth elements is also an important area of research. A high concentration of Ni is frequently used in polar steel materials as it can enhance toughness at low temperatures. For example, the Ni content in the low-temperature steel of FH36 grade is around 0.80 wt.%. WISCO (Wuhan Iron and Steel (Group) Company) has developed WQ960E (this is their company’s unique steel grade; Q960 represents the yield strength value in MPa, and E represents the quality grade), which exhibits an even higher nickel content of 2.0 wt.%. To guarantee that the desired low-temperature toughness characteristics are present in the steel used in polar regions, Ni can be added as it enhances the low-temperature fatigue properties [79]. Adding Ni also increases corrosion resistance in steel materials intended for polar environments. Besides Ni, Mn is also commonly added to steel. However, Mn readily forms strip MnS inclusions when combined with S in steel. This adversely affects the low-temperature toughness of steel and increases the susceptibility of the material to stress corrosion cracking. Hence, Mn addition generally requires the enhancement of the steel smelting technology to decrease the S content in steel. Alternatively, adding Ti and Cr to steel can facilitate the spheroidization of MnS, which can enhance the resistance to stress corrosion cracking [80]. Incorporating the appropriate amount of Nb in steel may not only refine the structure and enhance toughness but also increase the nucleation rate, quantity, and strengthening impact of precipitates. These changes can, in turn, effectively enhance the yield strength of steel and reduce its ductile-to-brittle transition temperature [81]. Introducing Nb results in a smoother corroded surface and denser corrosion products, which can decrease the degree of corrosion [82]. Incorporating Cr, Mo, and N can enhance the pitting resistance of steel materials. This makes them particularly well-suited for applications in corrosive environments that contain Cl^−^, such as seawater. Incorporating Cr and Mo into steel materials can enhance their resistance to wear and corrosion. This improvement ensures the safety and longevity of polar steel materials. The application of high-chromium steel in marine environments is often preferred due to its high wear and corrosion resistance, which is attributed to the formation of chromium carbide precipitates resulting from the interaction between Cr and C present in the steel. The presence of these precipitates increases the hardness and wear resistance. Additionally, Cr can produce a durable passive film on the surface and enhance the resistance of the surface to corrosion. However, the accumulation of chromium carbide at the grain boundary in high-chromium steel should be prevented because it can increase the brittleness of the grain boundary, decrease corrosion resistance, increase the susceptibility to intergranular corrosion, and lead to intergranular stress corrosion cracking. Thus, excess chromium carbide can act as the primary source of stress corrosion cracking. A comprehensive and rigorous investigation of the mechanism underlying the influence of rare earth elements on the low-temperature mechanical properties and corrosion properties of steel is extremely important. The above research endeavor contributes to the advancement of polar steel materials. The application of rare earth elements was found to enhance the resistance to low-temperature brittleness and improve its mechanical properties [83]. Rare earth elements also strongly affect the corrosion process [84,85,86]. The impact of incorporating various alloy elements into steel does not follow a simple additive relationship. Thus, a thorough assessment of the mechanism underlying the influence of multiple elements on steel can help in determining a more efficient alloy-addition strategy to enhance the overall performance of iron and steel. The investigation of iron and steel smelting, forming, heat treatment, processing, and related aspects needs to be emphasized when using alloying technology. The application of these technologies can also influence how the characteristics of the material are presented.

#### 2.3.2. Coating

A protective coating can effectively mitigate the corrosion of metal materials. However, in polar environments, characterized by extremely low temperatures and frequent storms and snowstorms, the exposed surfaces of components above the polar sea surface accumulate a thick layer of ice and snow. This accumulation can adversely affect the functionality of the components and may lead to damage and accidents. In the unique polar environment, the application of conventional coatings can decrease compactness, reduce adhesion, and cause self-embrittlement. The research and development of coatings for polar ships are thus quite limited [87,88]. Only a few enterprises, such as Jordan Marathon IQ and Akzo Nobel International Intershield163Inerta160 in the Netherlands, use pure epoxy technology and can make such coatings. The polar marine coatings Permax and Hempdur Multi-Strength GF35870 are primarily reinforced with glass fiber/scale. These coatings have high wear resistance and a low friction coefficient at low temperatures.

#### 2.3.3. Cathodic Protection

Cathodic protection technology is commonly used for mitigating corrosion in marine environments. It is grounded in the principles of electrochemical thermodynamics. The cathodic protection technology used in marine environments can be categorized into two main types: sacrificial anode cathodic protection and applied current cathodic protection [89,90,91,92]. Sacrificial anodes composed of Mg, Zn, Al, and their respective alloys are extensively used in the field of marine engineering. The application of cathodic protection technology in polar environments is in the research and development phase. Several challenges have hindered the implementation of cathodic protection using sacrificial anodes in polar environments. These challenges primarily arise from the presence of polar ice and snow cover, which can impede the anodic corrosion process and, subsequently, affect the electrochemical activity of the sacrificial anode. Consequently, the effectiveness of the sacrificial anode in preventing corrosion is reduced. The application of cathodic protection needs to be further studied and optimized based on the power supply equipment and the auxiliary anode within the cathodic protection system. The use of iron and steel materials in the polar region is influenced by complex and challenging environmental conditions, which result in distinct variations in the self-corrosion potential. To prevent the corrosion or hydrogen charging of protected iron and steel materials resulting from inadequate cathodic protection design, a systematic study needs to be conducted on the positioning and sizing of the sacrificial anode and the application of cathodic potential. This study should be performed according to specific environmental requirements.

## 3. Existing Problem

### 3.1. Low-Temperature Toughness

Expanding marine resource exploration in shallow water, abyssal sea, and polar high mountain areas necessitates the use of marine engineering steel with excellent low-temperature toughness. This is crucial for ensuring the structural safety of facilities in the Arctic region, where temperatures can drop as low as −70 °C [18]. In addition to inherent loads, such as wind loads, wave loads, ocean current loads, and seismic loads, external loads must also be considered in extremely cold weather. The structural design and material selection of the components must be able to effectively withstand the harsh conditions prevalent in this environment.

The catastrophic fracture that occurred on the Titanic 110 years ago was a result of the inadequate fracture resistance of the steel used at low temperatures. This was due to the high yield ratio and low safety level of the steel used in the ship. The material lacked significant plastic deformation capability before fracture. While a tragic event, it has spurred advancements in fracture mechanics, materials science, and technology. Presently, ship steel plates need to possess excellent mechanical properties, good weldability, cold bending ability, and seawater corrosion resistance simultaneously.

**Figure 6:**
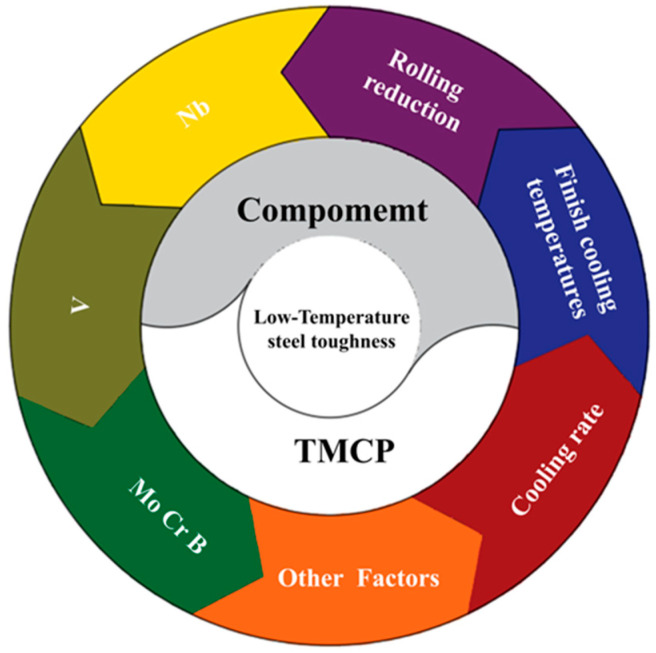
Schematic diagram of low-temperature toughness optimization of low-temperature steel. The typical factors affecting the low-temperature toughness properties of pipeline steel are shown, including composition (Nb, V, Mo, Cr, and B) and process parameters (finish cooling temperature, cooling rate, and rolling reduction).

Common methods for strengthening the low-temperature toughness of polar steel primarily focus on material composition and processing technology (Figure 6). Controlled rolling and cooling technology, as well as ultrasonic surface rolling, are utilized to shape and deform materials while attempting to reduce residual stress. These processes effectively leverage the fine grain strengthening and precipitation strengthening of metal materials.

Various elements have different effects on alloys. For example, Nb can refine austenite grains and deformed austenite grains by impeding grain boundary migration during the steel TMCP process, ultimately refining the microstructure of the material, enhancing its toughness and strength [93,94,95,96,97,98]. Alloy element V can improve the low-temperature toughness of welded steel and compensate for the lack of Nb precipitation strengthening. However, V is typically not used alone in alloy design since it possesses a stronger precipitation effect but a weaker grain refinement effect. Mo mainly enhances solute resistance and affects the precipitation activity of other alloy elements (V, Nb, Ti, etc.) in low-temperature steel. Additionally, Mo raises the activation energy of carbon diffusion in austenite, thereby reducing the dispersion coefficient of carbon and inhibiting the formation of proeutectic ferrite. It is worth noting that increasing the content of Mo may improve the alloy’s strength, but its toughness will decrease [99,100,101,102,103]. Cr, known as a ferrite stabilizer, enhances the strength of steel by forming chromium carbide.

The influence of different microstructures of materials on low-temperature mechanical properties is also significant. The adoption of a dual-phase structure composed of hard and soft phases in polar steel can provide greater resistance to deformation. In multiphase structures, the soft phase ensures plasticity while the hard phase ensures strength, achieving a good balance between strength and plasticity. Improving the hardness difference between the soft and hard phases can effectively reduce the yield strength ratio. However, if the hardness difference is too large, microcracks are prone to initiate and propagate at low temperatures, which is detrimental to toughness. Ferrite+pearlite type steel exhibits a distinct natural yield point but has poor resistance to deformation in pipelines. On the other hand, pipeline steel with a mixed structure of bainite (acicular ferrite) and ferrite possesses a dome-shaped stress-strain curve, offering high resistance to deformation.

When considering the adoption of these methods, attention should be given to both economic and environmental benefits. Polar regions have limited human intervention and fragile natural ecological environments. Therefore, it is essential to select environmentally friendly materials whenever possible. Additionally, cost reduction is an important focus when implementing these methods, and research efforts should be directed towards obtaining low-temperature steel grades that meet demand in a more cost-effective manner.

### 3.2. Corrosion in the Polar Region

**Figure 7:**
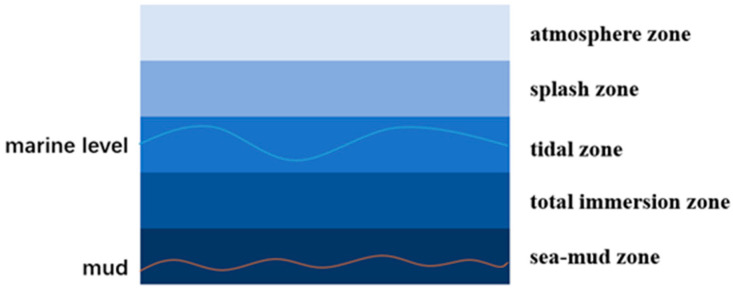
Corrosion area of traditional marine environment.

Corrosion is a problem not only for polar marine steel but also for traditional marine steel. The traditional corrosion zone of the marine environment can be categorized into five distinct areas based on spatial variations. These areas include the atmosphere area, splash area, tidal area, full immersion area, and sea-mud area (Figure 7). The atmospheric environment is susceptible to mold corrosion due to a combination of factors such as salt spray, rainfall, humidity, temperature, sunlight radiation, and other conditions that favor mold growth. Similarly, the splash area experiences corrosion due to an ample supply of oxygen and the alternating wet and dry conditions. In the intertidal zone, oxygen distribution is uneven, leading to a disparity in oxygen concentration between the upper and lower water surfaces. This difference gives rise to an oxygen concentration gradient, similar to a concentration cell. The corrosion rate of the oxygen concentration cell is relatively low when the cathode area is protected [91,104,105]. The corrosion rate in the fully immersed area is influenced by various factors, such as the salt content, pressure, dissolved oxygen, water temperature, and presence of sea life. Additionally, the corrosion rate can vary with changes in temperature and seawater depth. The presence of a large population of anaerobic microorganisms is responsible for the formation of the final sea-mud region, which occurs due to anaerobic microbial corrosion. In contrast to the conventional marine environment, the polar environment is characterized by its extreme nature, featuring unique conditions, including low temperatures, high salinity, strong winds, high waves, storms, snow, ice floes, and polar nights.

In the polar region, the temperature above the ocean is low throughout the year, and the distribution of humidity in the atmosphere is uneven [106]. When humidity is low, the supercooled salt spray exhibits the formation of loose, opaque, white granular structures known as sediment rime, as well as the development of thin fog frost upon contact with the steel surface. When the atmospheric humidity is high, the interaction between small droplets in the atmosphere and the steel surface results in the formation of denser rime ice on the steel surface. These findings indicate that the presence of a liquid film on the steel surface for a long time can be attributed to the condensation-melting mechanism of ice and snow. This process, in turn, facilitates and accelerates the localized corrosion of polar steel [107]. The steel surface, characterized by alternating dry and wet conditions, is influenced by factors such as polar day and night cycles, seasonal variations, and solar irradiation. The polar marine atmosphere experiences strong wind patterns, often leading to adverse weather conditions, such as storms and snowfall. These climatic factors contribute to particle erosion and corrosion on the surface of steel materials in the polar atmosphere. The high wind speeds also facilitate the diffusion of depolarizing agents. The presence of particles in the wind can influence the protective corrosion products or coatings on steel surfaces, thus increasing the likelihood and severity of erosion and corrosion.

The primary difference between the polar environment and the conventional marine environment is the presence of polar ice and snow. The extent of polar snow cover can vary based on seasonal and regional factors. When temperature increases, drift ice gets displaced, and ice and snow thaw, resulting in the formation of ice-free or ice-breaking regions in polar snow and ice regions. When polar vessels break ice, they generate icebreaking zones due to the interaction between their hulls and the snow and ice. Steel materials located in areas prone to polar ice-water erosion are frequently exposed to a mixed environment of ice and water [2]. The polar ice-water erosion area can be further categorized into distinct regions based on the different mixing ratios of ice, snow, and seawater. These regions include the ice-filled area, the icebreaking area, and the ice-free area [108].

In the ice-filled area, the corrosion of polar steel in ice-covered regions is primarily influenced by factors such as sea ice salinity, porosity, interstitial water, microorganisms, and other relevant variables [109,110]. Polar steel materials can develop concentration cells due to the non-homogeneous distribution of factors that influence the corrosion process in ice and snow, leading to uneven corrosion [111].

While breaking ice, the corrosion of polar steel materials can be characterized as a corrosion process involving a solid–liquid two-phase flow. In such cases, the corrosion process becomes complex and severe. Regarding the ice-water erosion corrosion of DH series low-temperature steel, at lower rotational speeds, corrosion plays a dominant role. However, as the rotational speed increases, the combined effect of corrosion and erosion wear becomes more pronounced. This is evident when the number and depth of erosion–corrosion pits on the steel sample surface increase. A higher ice-to-water ratio leads to more severe corrosion [112]. The degradation of polar steel materials in the fragmented ice region is influenced not only by factors such as the ratio of ice to water and the velocity of scouring but also by the variation in the size, shape, and angle of scouring of the sea ice. In the icebreaking zone, the salinity of seawater increases as a result of icing and salt precipitation. This leads to a noticeable disparity in the salt concentration between the ice–snow in the icebreaking zone and the surrounding seawater. The corrosion of polar steel materials in this area may exhibit uneven patterns due to different levels of salinity. During the icebreaking operation, an icebreaking area is created, where the icebreaker navigates. As the icebreaker moves through this area, the steel materials of its hull are eroded due to ice and water. When the icebreaker breaks the ice, the steel materials experience fatigue due to the impact of ice and snow. The steel of the icebreaker is commonly affected by fatigue corrosion caused by ice and water. Thus, the fatigue corrosion of icebreaker steel occurs commonly in this region. The polar tide is hindered by the presence of crushed ice, which reduces the impact of this phenomenon on the corrosion of polar steel materials in the icebreaking area [113,114,115,116].

In ice-free regions, where ice and snow cover are absent and oxygen is abundant, corrosion mass transfer can occur more easily compared to environments with ice and snow. The polar region shows prominent wind patterns and strong atmospheric conditions, which increase the size of waves. The presence of diurnal tides further facilitates the formation of large waves and a significant tidal range within this geographical zone. The polar steel materials found in this region can withstand the environmental effects of the spray splash zone and tidal range area, which are similar to those of the conventional marine environment. However, the temperature in this region is relatively low, and any water present on the steel surface due to spatter or tidal range can undergo a phase transition at these lower temperatures. Hence, the corrosion of polar steel materials in this region exhibits similarities not only to the corrosion observed in the spray splash zone and the tidal range zone of conventional marine environments but also to the dry and wet corrosion resulting from the cyclic phase transition of sea-water on the surface of steel. The corrosion of polar steel is strongly affected by the ratio between dry and wet conditions. As the dry-to-wet ratio increases, the thickness of the layer of rust on the surface of carbon steel decreases. This reduction is accompanied by an increase in the content of γ-FeOOH and oxygen in the corroded products, whereas the content of Fe_3_O_4_ decreases. Consequently, the severity of corrosion intensifies [117]. The corrosion experienced by polar marine steel vessels in ice-free regions due to seawater erosion is a highly significant form of degradation [118].

Besides the aforementioned issues, the polar marine environment has a large community of identified and unidentified microorganisms [119]. The investigation of the corrosion of polar steel materials in the polar atmosphere is extremely important and requires immediate attention. The presence of subaquatic volcanoes in the polar regions, resulting from tectonic activity, gives rise to hydrothermal regions within the polar ocean submergence zones. In these regions, seawater temperatures frequently reach up to 400 °C [42,120,121,122,123]. Therefore, the criteria for the corrosion characteristics of steel materials are more rigorous.

## 4. Suggestions for Development

To ensure the suitability of polar steel materials for application in polar environments, the satisfaction of the mechanical property requirements needs to be prioritized during research and development. In polar environments, polar steel needs to have certain characteristics to be used as a structural material. These include a low brittle-ductile transition temperature, high yield strength, exceptional low-temperature crack arrest capability, favorable weldability, and excellent wear resistance. These properties ensure the performance and durability of polar steel under challenging conditions such as low temperatures, serviceable corrosion resistance, strong winds, high waves, snowstorms, and ice floes [14].

In this article, based on many previous research results, to address existing limitations, we proposed future development directions of polar and low-temperature steel:

1. In steel production, the control of chemical composition and TMCP parameters is essential for achieving the desired special performance in specific usage environments. For instance, ultrafine grains can be achieved through controlled rolling, controlled cooling, and intricate multi-stage thermoplastic processing. This micro/nano scale structure enhances the low-temperature resistance and strength of low-alloy structural steel. Despite being a widely recognized textbook principle, its significance should not be overlooked.
2. Establish a comprehensive database for polar steel to facilitate research in this field. In recent years, extensive data has been produced on the composition and performance of polar steel. However, polar steel lacks a grading system and high-quality dataset. The database should encompass the composition, TMCP parameters, and performance characteristics. Furthermore, standards and specifications for steel plates, profiles, deck machinery, slurry, and core shaft system components for polar vessels need to be developed and refined.
3. Combine machine learning with existing polar steel data. Machine learning employs deep learning and other techniques to analyze and model the composition, microstructure, and process parameters of steel materials, facilitating performance prediction and optimization. By integrating various computational models with experimental research, we can enhance our comprehension of the relationship between the microstructural characteristics and properties of pipeline steel.
4. Develop the manufacture of green and sustainable polar steel. Only by achieving green sustainability in the production and use of polar steel can we further develop the polar regions with the application of polar steel. Therefore, reducing the impact of polar steel on the polar environment while simultaneously enhancing its necessary performance will be the primary focus of future research on polar steel.

## Data Availability

No new data were created or analyzed in this study.
